# Application of the integrated gateway model on child nutrition behaviors in Niger: An exploratory analysis

**DOI:** 10.1371/journal.pone.0297466

**Published:** 2024-04-01

**Authors:** Leanne Dougherty, Chaibou Dadi

**Affiliations:** 1 Population Council, Washington, DC, United States of America; 2 Conception Etudes Suivi Evaluation Appuis Formation, Niamey, Niger; Ateneo de Manila University Ateneo School of Medicine and Public Health, PHILIPPINES

## Abstract

**Objective:**

To identify potential gateway factors and behaviors that are associated with infant and young child feeding (IYCF) practices in the Maradi and Zinder regions of Niger through application of the Integrated Gateway Model.

**Methods:**

We analyzed data from 2,727 married women of reproductive age including details on child feeding practices for their 2,551 children between the ages of 0 to 23 months. We assessed the association of three gateway behaviors (i.e., any antenatal care, facility delivery, and communication on nutrition practices) and gateway factors (i.e., behavioral determinants, exposure to information, decision-making agency, and woman’s group participation) on four IYCF practices (i.e., early initiation of breastfeeding, exclusive breastfeeding, minimum meal frequency, and minimum dietary diversity) while controlling for age, parity, educational attainment, and household wealth.

**Results:**

We found antenatal care was associated with exclusive breastfeeding of children 0–5 months [adjusted odds ratio (aOR): 1.17 (95% confidence interval (CI): 1.03–1.33)], and minimum meal frequency of children 6–23 months [aOR: 1.10 (95% CI: 1.03–1.17)], and facility delivery was associated with early initiation of breastfeeding among children 0–5 months [aOR: 2.08 (95% CI: 1.39–3.12)]. We found negative associations with exclusive breastfeeding and communication on nutrition practices with husbands, family/friends, and health workers. Exposure to nutrition messages through radio, women’s groups participation, and with health workers was positively associated with minimum dietary diversity. Self-efficacy was positively associated with both early initiation of breastfeeding, exclusive breastfeeding among children 0–5 months and minimum dietary diversity among children 6–23 months. Women’s agency was positively associated with early initiation of breastfeeding.

**Conclusion:**

The association of antenatal care and facility delivery with child nutrition outcomes suggest intervening before a woman becomes pregnant or early in her pregnancy could improve nutrition outcomes. Programs strengthening the continuum of care should identify gateway behaviors to maximize adoption of priority health behaviors.

## Introduction

In Niger, nearly 83,000 children under the age of five died in 2018 [1]. Malnutrition and suboptimal breastfeeding are leading contributors to child deaths [2]. According to the 2012 Demographic and Health Survey in Niger, nearly 44% of children under the age of five were stunted, breastfeeding in the first hour after birth was 53%, and exclusive breastfeeding ended prior to six months for three quarters of children [3]. Several factors contribute to child malnutrition including food insecurity, feeding and caregiving practices, and access to health services [4]. Caregivers’ ability to practice optimal feeding practices, including the amount needed to feed a child and the food types for a balanced diet, are factors that contribute to the deterioration of a child’s nutritional status. As such, interventions aimed to address barriers to appropriate infant and young child feeding (IYCF) practices are needed to improve child nutrition outcomes in settings such as Niger [5].

Previous studies have found that integrating infant and young child nutrition counseling during antenatal care (ANC), postnatal care, family planning or child consultation visits can be an effective approach to improving infant and young child feedings in some settings [6–8]. However, findings from a scoping review and narrative analysis published in 2023 found that strategies to improve interpersonal communication along the continuum of maternal and newborn care centered around information sharing and that women, their partners and families could benefit from a more comprehensive, integrated communication strategy [9]. Integrated social and behavior change (SBC) programming is an evidence-driven approach that aims to affect key behaviors and social norms by addressing their individual, social, and structural determinants (factors). The “Integrated Gateway Model” provides a framework to guide interventions aimed at supporting continuum of care by situating positive behaviors in reproductive, maternal, and child health within an integrated SBC perspective thereby supporting the development of a more comprehensive, integrated communication strategy [10].

**Fig 1:**
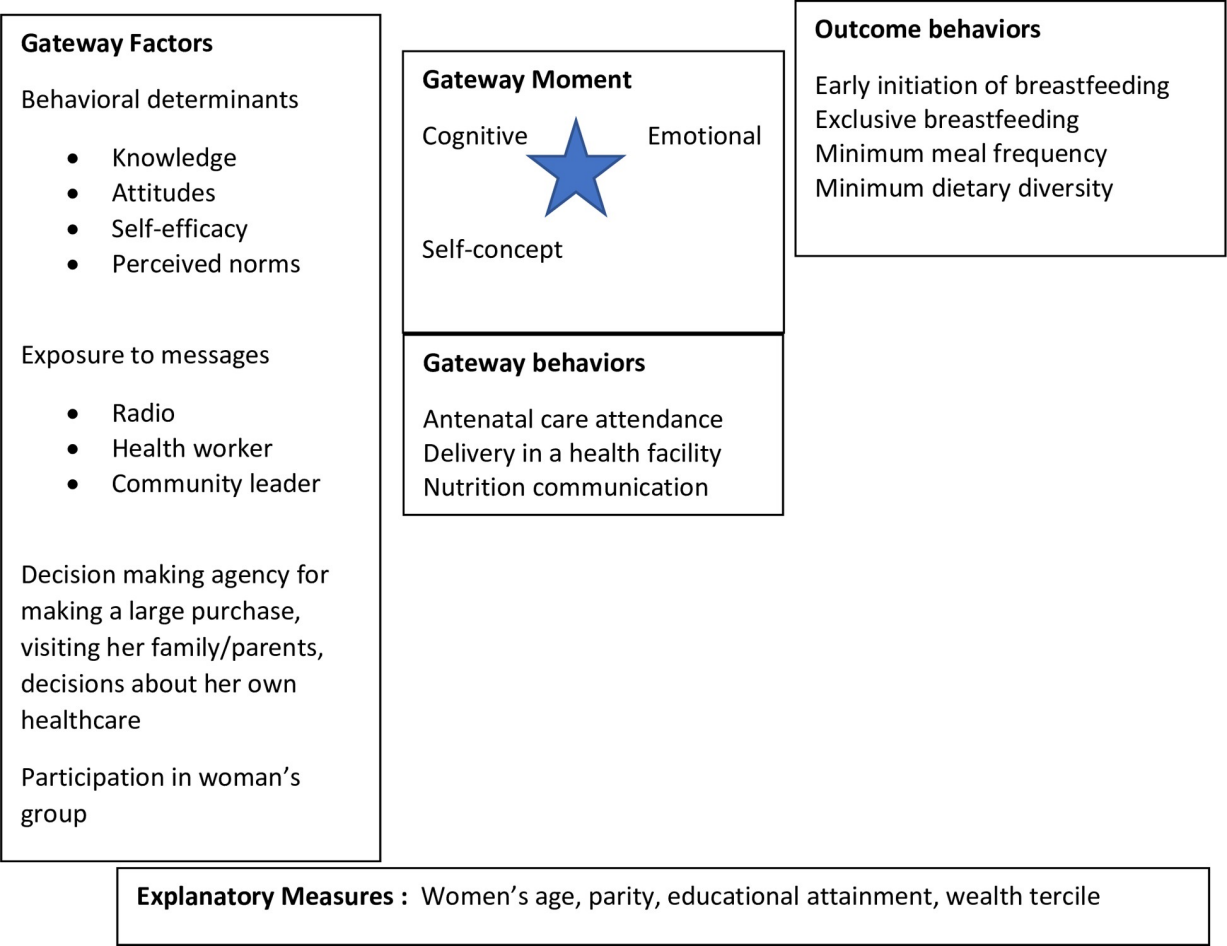
Integrated gateway model application to child health nutrition behaviors (Adapted from Schwandt 2015) [12].

The present study aims to further our understanding of how gateway behaviors which are actions that precede or influence future behaviors [11] can be leveraged to influence behavior change of IYCF practices in Niger. Our analysis is guided by the Integrated Gateway Model (Fig 1) [10]. In the integrated gateway model, a gateway behavior takes place within a gateway moment. Gateway moments refer to key transitional points in life (e.g., pregnancy or birth) when individuals may be receptive to new information and motivated to make positive health changes. These life transitions might be developmental, situational (such as pregnancy or marriage), health-related, or illness-related and are characterized by three elements: cognitive, emotional, and self-concept. During this time, one or more gateway factors operate to influence the gateway behavior. A gateway factor refers to the context, attributes, or conditions that facilitate behavior change and might have a positive or negative influence on subsequent behaviors. Previous studies aimed at understanding the effects of gateway behaviors on health outcomes have been conducted in high-income countries and have focused on healthy eating and physical activity [12,13]. Few studies have explored the gateway concept in other health areas and especially among low-income countries. With this quantitative exploratory analysis, we identify potential gateway factors and behaviors that are associated with IYCF practices using recently collected sub-national data to inform integrated SBC programming in the Sahel region.

## Materials and methods

### Study design and setting

**Fig 2:**
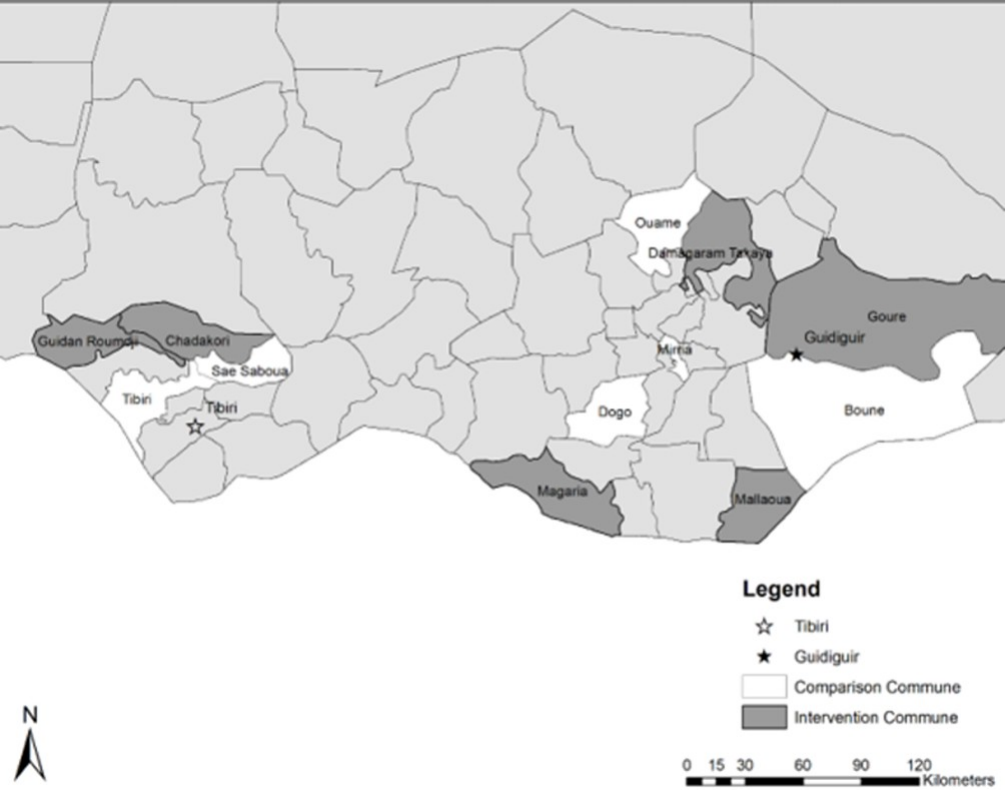
Map of Niger and RISE II study areas.

This study drew from the U.S. Agency for International Development’s (USAID’s) Breakthrough RESEARCH integrated SBC evaluation of the Resilience in the Sahel Enhanced II (RISE II) program. The RISE II program (2018–2023) addresses maternal, newborn and child health, family planning, and water, sanitation, and hygiene through an integrated SBC strategy which aims to address common underlying barriers to health-seeking behaviors. The study used the endline household survey collected in February 2023 in Niger’s Maradi and Zinder regions (Fig 2).

Maradi includes the city of Maradi, the second largest in Niger, but overall is quite rural. Zinder is very rural and extends north into the Sahel. Households in these districts rely on rainfed agriculture and pastoralism for their livelihoods.

### Sampling

We applied a three-stage stratified sampling procedure. In the first stage, we randomly selected six intervention communes from the 18 intervention communes and six comparison communes (four in Zinder and two in the Maradi region) based on similar demographic characteristics and healthcare accessibility. In the second stage, we listed all enumeration areas (EAs) identified in the 2012 general census by commune in each of the randomly selected communes. We then used probability proportional to size to select EAs per commune starting at a random point and then systematically selecting areas using a fixed sampling interval. In total, we sampled 40 EAs for each group, stratified by commune. In the third stage, we enumerated all households in each of the randomly selected EAs with eligible women (married women of reproductive age [MWRA] between 15 and 49 years of age).

The survey reached 2,727 MWRA and collected data for 652 children 0–5 months and 1,899 children between the ages of 6 to 23 months for a total of 2,551 children 0–23 months. This MWRA sample size is based on a minimum detectable difference for evaluation purposes of 6–11% points in the priority indicators (e.g., skilled birth attendance) between study groups, with 80% power to detect a difference and α = 0.05. From households with eligible women, we randomly selected 34 households per EA to account for a 10% nonresponse rate and interviewed about 30 women between 15 and 49 years of age. The response rate was nearly 100%.

### Data collection

Data collection was conducted by a Niger-based research partner, Conception Etudes Suivi Evaluation Appuis Formation, and administered in the local languages (e.g., Hausa). The research partner recruited interviewers locally and trained them on techniques to ensure privacy and confidentiality as well as to minimize bias by using historical and recent events to facilitate recall. Interviewers administered surveys using tablets and uploaded data to a secure server.

### Measures

#### Outcome measures

Nutrition outcomes included breastfeeding practices and child feeding which were constructed according to the Demographic and Health Survey Guide to Statistics [14].

- Early initiation of breastfeeding: Percentage of children between 0 and 5 months who received breast milk immediately after birth
- Exclusive breastfeeding: Percentage of children between 0 and 5 months who received only breast milk in the 24 hours preceding the survey
- Minimum meal frequency: Proportion of breastfed and non-breastfed children 6 to 23 months of age who receive solid, semisolid, or soft foods (but also including milk feeds for non-breastfed children) the minimum number of times or more. Note: For breastfed children, the minimum number of times varies with age (2 times if 6 to 8 months and 3 times if 9 to 23 months). For non-breastfed children the minimum number of times does not vary by age (four times for all children 6 to 23 months)
- Minimum Dietary Diversity: Proportion of children 6 to 23 months of age who receive foods from four or more food groups over the 24-hour recall period.

#### Explanatory measures

Explanatory measures included mothers age, parity, educational attainment, and household wealth. Household wealth terciles were calculated based on a series of survey questions assessing household asset ownership using the Equity Tool methodology [15].

#### Gateway factors

Gateway factors included behavioral determinants (e.g., knowledge, attitudes, self-efficacy and perceived norms for breastfeeding and child feeding practices), exposure to breastfeeding and child feeding messages in the three months prior to the survey, a woman’s household decision-making, and participation in a woman’s group which targeted women from pregnancy through the first two years of a child’s life.

#### Gateway behaviors

Gateway behaviors included any ANC use, delivery in a facility and communication with partners, family/friends, or health providers related to nutrition.

Table 1 provides details on variable construction for the explanatory measures, gateway factors and gateway behaviors used in the analysis.

**Table 1:** Description of variables used in application of integrated gateway model.

Type	Variable name	Definition
Explanatory measure	Age	Categorical: Percentage of MWRA who reported their current age and grouped (15–24 years, 25–34 years, 35–49 years)
Explanatory measure	Parity	Categorical: Percentage of MWRA who reported number of living children and grouped (1–2 children, 3–4 children, 5–6 children, and 7+)
Explanatory measure	Educational attainment	Binary: Percentage of MWRA who reported to have ever gone to school.
Explanatory measure	Household wealth tercile	Categorical: Percentage of MWRA who reported household assets constructed in wealth terciles (poorest, middle, richest)
Gateway factor	Knowledge	Early initiation and exclusive breastfeeding: • Binary: Percentage of MWRA who state it is it healthy for a woman to give only breast milk for the first 6 months Minimum meal frequency: • Binary: Percentage of MWRA who reported a child 6–23 months should eat 4 or more meals each day Minimum acceptable diet: • Binary: Percentage of MWRA who reported that the number of different types of food a child 6–23 months should eat a day is 4 or more
Gateway factor	Attitude	Early initiation and exclusive breastfeeding: • Binary: Percentage of MWRA who agree if a baby is exclusively breastfed for 6 months, he/she is less likely to be sick Minimum meal frequency: • Binary: Percentage of MWRA who agree providing children 4 meals a day ensures they have strength Minimum acceptable diet: • Binary: Percentage of MWRA who agree children who eat a variety of foods are less likely to get sick
Gateway factor	Self-efficacy	Early initiation and exclusive breastfeeding: • Binary: Percentage of MWRA who agree giving only breast milk to the baby for the first 6 months is not difficult at all Minimum meal frequency: • Binary: Percentage of MWRA who agree giving a child a meal 4 times a day is not difficult at all Minimum acceptable diet: • Binary: Percentage of MWRA who say giving a child a minimum of 4 or more different types of food a day is not difficult at all
Gateway factor	Perceived norms	Early initiation and exclusive breastfeeding: • Binary: Percentage of MWRA who agree people in this community think is it healthy for a woman to give her baby only breast milk for the first 6 months Minimum meal frequency: • Binary: Percentage of MWRA who believes the number of meals people in community think a child 6–23 months should eat each day is 4 or more Minimum acceptable diet: • Binary: Percentage of MWRA who believes number of different types of food people in the community think a child 6–23 months should eat a day is 4 or more
Gateway factor	Exposure to nutrition messages	Binary: Percentage of MWRA who had heard or seen a message related to breastfeeding or young child nutrition from the radio, health worker, or community event in the past 3 months
Gateway factor	Women’s involvement in decision making	Binary: Percentage of MWRA who responded that either she made the decision or that she and her partner jointly makes the decision for all three decisions: 1) making a large purchase, 2) visiting her family/parents, and 3) decisions about her own healthcare
Gateway factor	Participation in woman ‘s group	Binary: Percentage of MWRA who belong to a women’s community group
Gateway behavior	Any antenatal care	Continuous: Percentage of MWRA who have given birth in the 5 years preceding the survey who received ANC visits for their last birth
Gateway behavior	Facility delivery	Binary: Percentage of MWRA who have given birth in the 5 years preceding the survey who delivered in a facility for their last birth
Gateway behavior	Primary person you speak with regarding your child’s nutrition	Categorical: Percentage of MWRA who spoke with 1) husband/partner, 2) family member, 3) health provider, or 4) no one about child’s nutrition

### Analysis

We first conducted a descriptive analysis to assess measures in the integrated gateway model followed by multivariate logistic regression models to explore the extent to which gateway factors and behaviors were associated with breastfeeding and child nutrition outcomes. The four main nutritional outcomes explored were: 1) initiation of breastfeeding immediately following delivery among children 0–5 months; 2) exclusive breastfeeding of children 0–5 months; 3) minimum meal frequency among children 6–23 months and 4) minimum dietary diversity among children 6–23 months. We controlled for age, parity, educational attainment, and household wealth terciles. We constructed three models for each gateway behavior. The models were run separately due to correlations between the behaviors. The first model tested the association of any ANC on the IYCF outcomes, the second model tested the association of facility delivery, and the third model tested communication related to nutrition. In each model, we included behavioral determinant gateway factors (e.g., knowledge, attitudes, self-efficacy, and perceived norms). Behavioral determinant gateway factors varied by nutritional outcome. Based on data available in the survey, there was a single set of behavioral determinant variables for breastfeeding while there were specific behavioral determinant variables for minimum meal frequency and minimum dietary diversity. Therefore, we applied the behavioral determinant gateway factors specific to each behavior in the model. We also assessed exposure to information, household decision making and women’s group participation. Models were adjusted for standard errors by commune and EA to account for the sampling approach and study area. Analyses were completed using Stata version 16 [16].

## Results

A total of 2,727 MWRA participated in the survey. Approximately 50% of women were between the ages of 25 to 34 and over 80% had never attended school (Table 2). Over 50% of women interviewed had five or more children. Regarding the practice of gateway behaviors, most study participants received on average three ANC visits for their last birth and 48% had delivered in a health facility. More than two-thirds (66%) of study participants said their husband was the primary person they spoke with about their child’s nutrition. Among children 0–5 months, 59.1% were exclusively breastfed while 56.9% received breast milk within the first 24 hours after birth. Approximately 60% of children 6 to 23 months received the minimum meal frequency while only 10% received four or more meals. Gateway factor descriptive analyses are available in S1 and S2 Tables.

**Table 2:** Description of RISE II study sample.

	**Total n (%)**
**Characteristics among women of reproductive age** (N = 2,727)	
**Age**	
15–24 years	725 (26.7)
25–34 years	1,358 (49.8)
35–49 years	639 (23.4)
Missing	5 (0.2)
**Parity**	
1–2 children	576 (21.1)
3–4 children	695 (25.5)
5–6 children	684 (25.1)
7+ children	760 (27.1)
Missing	12 (0.4)
**Educational attainment**	
No education	2,245 (82.3)
Some formal schooling	482 (17.7)
**Household wealth terciles**	
Poorest	977 (35.8)
Middle	841 (30.8)
Richest	909 (33.3)
**Gateway behaviors among women of reproductive age** (N = 2,727)	
Mean ANC visits for their last birth (Standard Deviation)	3.1 (1.5)
Delivered in a facility for their last birth	1,300 (47.7)
Primary person the mother speaks with about child’s nutrition	
Husband/partner	1,811 (66.4)
Family member	373 (13.7)
Health provider	331 (12.1)
No one	212 (7.8)
**Nutrition outcomes among children 0–5 months** (N = 652) & children 6–23 months (N = 1,899)	
Proportion of children 0–5 months who initiated breastfeeding within the first 24 hours after birth	371 (56.9)
Proportion of children 0–5 months exclusively given breast milk in the 24 hours preceding the survey	385 (59.1)
Minimum meal frequency among children 6 to 23 months	1, 120 (59.0)
Dietary diversity among children 6 to 23 months	190 (10.0)

Table 3 presents multivariate associations between gateway factors and each of the three gateway behaviors (e.g., any ANC at last birth, facility delivery at last birth and communication on child nutrition) and breastfeeding outcomes. Findings from the logistic regression model examining these associations with early initiation of breastfeeding show that women who delivered in a health facility at their last birth are more likely to initiate early breastfeeding [adjusted odds ratio (aOR): 2.08 (95% CI: 1.39–3.12)]. A number of gateway factors were positively associated with early initiation of breastfeeding. Self-efficacy (i.e., belief that giving a baby food and liquids when he/she is six months of age is not difficult at all) was statistically significant in all three models [Model 1: aOR 2.06 (95% CI: 1.18–3.58); Model 2: aOR: 1.91 (95% CI: 1.12–3.25); Model 3: aOR: 2.15 (95% CI: 1.24–3.72)]. Exposure to breastfeeding messages in the last three months through community events [Model 1: aOR 3.55 (95% CI: 1.53–8.24); Model 2: aOR: 4.00 (95% CI: 1.71–9.34); Model 3: aOR: 4.41 (95% CI: 1.74–11.2)] and women’s decision making [Model 1: aOR 2.20 (95% CI: 1.25–3.86); Model 2: aOR: 2.27 (95% CI: 1.31–3.94); Model 3: aOR: 2.62 (95% CI: 1.42–4.83)] were also positively associated with early initiation of breastfeeding.

**Table 3:** Multivariate analysis of gateway model and breastfeeding outcomes among children 0–5 months, RISE II Niger, 2023^a^.

	Early initiation of breastfeeding (N = 652)			Exclusive breastfeeding (N = 652)		
	*Model 1* *OR (CI)*	*Model 2* *OR (CI)*	*Model 3* *OR (CI)*	*Model 1* *OR (CI)*	*Model 2* *OR (CI)*	*Model 3* *OR (CI)*
**Gateway Factors**						
*Breastfeeding behavioral determinants*						
Knowledge: State it is healthy for a woman to give only breast milk for the first 6 months (reference: greater than 6 months or don’t know)	1.30 [0.71–2.37]	1.36 [0.75–2.50]	1.50 [0.80–2.82]	1.50 [0.83–2.70]	1.59 [0.85–2.85]	1.58 [0.88–2.84]
Attitude: Agree if baby is exclusively breastfed for 6 months, he/she is less likely to be sick (reference: disagree or neutral)	0.69 [0.35–1.33]	0.64 [0.33–1.23]	0.72 [0.36–1.48]	0.77 [0.37–1.65]	0.82 [0.39–1.73]	0.77 [0.36–1.62]
Self-efficacy: Giving a baby food and liquids when he/she is 6 months of age is not difficult at all (reference: somewhat not difficult or very difficult)	2.06 [1.18–3.58]*	1.91 [1.12–3.25]*	2.15 [1.24–3.72]**	4.22 [2.86–6.23]***	4.37 [2.96–6.46]***	4.48 [2.97–6.75]***
Perceived norms: State people in this community think is it healthy for a woman to give her baby only breast milk for the first 6 months (reference: greater than 6 months or don’t know)	0.68 [0.42–1.10]	0.66 [0.41–1.07]	0.75 [0.46–1.22]	0.69 [0.37–0.98]*	0.62 [0.39–0.99]*	0.64 [0.40–1.04]
*Exposure to breastfeeding messages in last 3 months (reference*: *no exposure)*						
Radio	0.92 [0.37–2.24]	0.85 [0.35–2.07]	0.65 [0.27–1.57]	1.71 [0.76–3.82]	1.88 [0.84–4.20]	1.74 [0.77–3.93]
Health worker	1.30 [0.80–2.12]	1.26 [0.79–2.01]	0.92 [0.54–1.59]	1.33 [0.88–2.03]	1.49 [0.97–2.30]	1.40 [0.91–2.16]
Community event	3.55 [1.53–8.24]**	4.00 [1.71–9.34]**	4.41 [1.74–11.2]**	2.27 [1.18–4.35]*	2.19 [1.14–4.20]*	2.93 [1.45–5.92]**
*Genderdecision-making*						
Decides alone or jointly with partner (for purchases, visits, health seeking) (reference: partner decides)	2.20 [1.25–3.86}**	2.27 [1.31–3.94]**	2.62 [1.42–4.83]**	0.39 [0.20–0.73]**	0.37 [0.19–0.68]	0.44 [0.23–0.84]*
*Woman’s group participation (reference*: *no participation)*						
Participated in group	1.17 [0.72–1.90]	1.16 [0.70–1.91]	1.26 [0.78–2.05]	1.10 [0.65–1.87]	1.16 [0.67–2.01]	1.17 [0.67–2.03]
**Gateway Behaviors**						
Any ANC	1.11 [0.96–1.28]			1.17 [1.03–1.33]*		
Facility delivery (reference: no facility delivery)		2.08 [1.39–3.12]***			0.86 [0.55–1.36]	
Communication on child nutrition (reference: no one)						
Husband/partner			0.49 [0.27–0.90]*			0.27 [0.14–0.52]***
Health worker			3.15 [1.09–9.13]*			0.39 [0.18–0.83]*
Other family/friend			0.63 [0.28–1.42]			0.17 [0.08 = 0.36]***

^a^Model controls for woman’s age, parity, educational attainment, household wealth tercile; CI = confidence interval; OR = odds ratio.

*p < .0.05

***p<0.001.

Findings from the logistic regression model examining associations (Table 3) with exclusive breastfeeding show that women who discuss child nutrition with their husband, a health worker or family/friend were statistically significantly less likely to exclusively breastfeed than if they spoke with no one. Gateway factors associated with exclusive breastfeeding again included self-efficacy, which was statistically significant in all three models [Model 1: aOR 4.22 (95% CI: 2.86–6.23); Model 2: aOR: 4.37 (95% CI: 2.96–6.46); Model 3: aOR: 4.48 (95% CI: 2.97–6.75)].

Table 4 presents multivariate associations between gateway factors and each of the three gateway behaviors (e.g., any ANC, facility delivery, and communication on child nutrition) and child nutrition outcomes. Findings from the logistic regression model examining these associations with minimum meal frequency show that children whose mothers received any ANC at their last birth are more likely to receive the minimum meal frequency [aOR: 1.10 (95% CI: 1.03–1.17)]. Gateway factors associated with minimum meal frequency include knowledge (i.e., a child 6–23 months should eat 4 or more meals each day), which was statistically significant more likely in each of the three minimum meal frequency models [Model 1: aOR 1.88 (95% CI: 1.46–2.41); Model 2: aOR: 1.86 (95% CI: 1.45–2.39); Model 3: aOR: 1.84 (95% CI: 1.43–2.37)]. Exposure to nutrition messages in the last three months through a community event was negatively associated with minimum meal frequency in all three models [Model 1: aOR 0.67 (95% CI: 0.48–0.94); Model 2: aOR: 0.68 (95% CI: 0.48–0.95); Model 3: aOR: 0.70 (95% CI: 0.50–0.99)].

**Table 4:** Multivariate analysis of gateway model and nutrition outcomes among children 6–23 months, RISE II Niger, 2023^a^.

	Minimum Meal Frequency (N = 1,899)			Minimum Dietary Diversity (N = 1,747)		
	*Model 1* *OR (CI)*	*Model 2* *OR (CI)*	*Model 3* *OR (CI)*	*Model 1* *OR (CI)*	*Model 2* *OR (CI)*	*Model 3* *OR (CI)*
**Gateway Factors**						
*Minimum meal frequency behavioral determinants*						
Knowledge: A child 6–23 months should eat 4 or more meals each day (reference: fewer than 4 or more meals)	1.88 [1.46–2.41]***	1.86 [.145–2.39]***	1.84 [1.43–2.37}***			
Attitude: Agree providing children 4 meals a day ensures they have strength (reference: disagree or neutral)	0.88 [0.40–1.94]	0.93 [0.44–1.98]	0.90 [0.43–1.91]			
Self-efficacy: Give child a meal 4 times a day is not difficult at all (reference: difficult or somewhat difficult)	0.93 [0.76–1.14]	0.92 0.75–1.12]	0.91 [0.75–1.11]			
Perceived norms: Believes number of meals people in community think a child 6–23 months should eat each day is 4 or more (reference: fewer than 4 or more meals)	0.78 [0.61–0.98]*	0.77 [0.61–0.98]*	0.79 [0.63–0.99]*			
*Minimum dietary diversity behavioral determinants*						
Knowledge: Number of different types of food a child 6–23 months should eat a day is 4 or more (reference: fewer than 4 or more types of food)				1.07 [0.63–1.81]	1.09 [0.64–1.85]	1.09 [0.64–1.86]
Attitude: Agree children who eat a variety of foods are less likely to get sick (reference: disagree or neutral)				2.50 [0.72–8.67]	2.51[0.72–8.79]	2.56 [0.73–8.93]
Self-efficacy; Give child a minimum of 5 different types of food a day is not difficult at all (reference: somewhat difficult, or very difficult)				1.64 [1.09–2.46]*	1.65 [1.09–2.5]*	1.67 [1.11–2.50]*
Perceived norms: Believes number of different types of food people in the community think a child 6–23 months should eat a day is 4 or more (reference: fewer than 4 or more types of food)				1.68 [0.85–3.31]	1.68 [0.84–3.32]	1.80 [0.90–3.60]
*Exposure to nutrition messages in last 3 months (reference*: *no exposure)*						
Radio	1.43 [0.89–2.27]	1.51 [0.94–2.42]	1.47 [0.93–2.33]	3.22 [1.71–6.05]***	3.401.82–6.35]***	3.30 [1.80–6.04]***
Health worker	1.14 [0.90–1.45]	1.19 [0.94–1.50]	1.08 [0.79–1.48]	3.13 [2.00–4.91]***	3.23 [2.06–5.08]***	3.0 [1.75–5.11]***
Community event	0.67 [0.48–0.94]*	0.68 [0.48–0.95]*	0.70 [0.50–0.99]*	0.36 [0.18–0.70]**	0.36 [0.18–0.71]**	0.39 [0.18–0.88]*
*Gender decision-making*						
Decided alone or jointly with partner (for purchases, visits, health seeking) (reference: partner decides)	1.03 [0.75–1.40]	0.99 [0.72–1.35]	1.04 [0.88–1.80]	1.06 [0.59–1.89]	0.99 [0.55–1.79]	1.13 [0.634–2.03]
*Woman’s group participation (reference*: *no participation)*						
Participated in group	0.98 [0.72–1.33]	1.03 [0.76–1.39]	1.07 [0.69–1.67]	2.19 [1.49–3.22]***	2.29 [1.57–3.33]***	2.26 [1.55–3.30]***
**Gateway Behaviors**						
Any ANC	1.10 [1.03–1.17}**			1.10 [0.97–1.26]		
Facility delivery (reference: no facility delivery)		1.02 [0.83–1.26]			1.04 [0.66–1.64]	
Communication on child nutrition (reference: no one)						
Husband/partner			1.13 [0.73–1.73]			0.80 [0.29–2.23]
Health worker			1.42 [0.84–2.39]			0.96 [0.31–2.96]
Other family/friend			1.06 [0.64–1.78]			0.56 [0.11–2.75]

^a^Model controls for woman’s age, parity, educational attainment, household wealth tertile; CI = confidence interval; OR = odds ratio.

***p<0.001

**p <0.01

*p<0.05.

Findings from the logistic regression model examining associations (Table 4) with minimum dietary diversity did not find statistically significant associations with gateway behaviors. Gateway factors positively associated with minimum dietary diversity included participation in a woman’s group [Model 1: aOR 2.19 (95% CI: 1.49–3.22); Model 2: aOR: 2.29 (95% CI: 1.57–3.33); Model 3: aOR: 2.26 (95% CI: 1.55–3.30)]. Exposure to nutrition messages in the last three months from the radio and a health worker, were all statistically significantly and positively associated with minimum dietary diversity in all three models while negative associations were observed with community events.

## Discussion

This study, guided by the integrated gateway model, examines the association of three gateway behaviors (i.e., any ANC, facility delivery, and communication on nutrition practices) and breastfeeding and child feeding practices in the Maradi and Zinder regions of Niger. A previous situational analysis of infant and young child nutrition policies and programmatic activities in Niger identified the need for improved monitoring and evaluation of IYCF programs to identify promising strategies for broader implementation and scale-up [17]. This paper contributes to this research priority by identifying potential gateway behaviors and factors that can be addressed through integrated SBC programs.

Consistent with previous studies, we found a positive association of ANC use and minimum meal frequency. However, we did not find an association between ANC use and other nutrition outcomes including breastfeeding and minimum dietary diversity. Early initiation of breastfeeding and facility delivery were also positively associated. This is not surprising as health workers present at birth are likely to encourage adoption of this breastfeeding practice given its health benefits to the mother and child following delivery. We did not see an association with facility delivery and exclusive breastfeeding, and child feeding practices, indicating that while mothers may not remember discussion on these matters six months after childbirth or there are other barriers to exclusive breastfeeding, it may also be a missed opportunity for health workers to promote positive IYCF behaviors at delivery. While we did not find a positive association with nutrition communication and IYCF outcomes (except for with early initiation of breastfeeding and communication with health workers), we did find that women’s communication with her husband/partner, health workers, and family/friends were not associated with child nutrition outcomes but were negatively associated with exclusive breastfeeding contrary to previous research [18]. Women in our sample were more likely to have had previous experience with breastfeeding given the parity levels among women interviewed. We considered that the negative association with speaking with someone about exclusive breastfeeding may be an effect modification related to high levels of parity, but this was not observed during our analysis (data not shown). Given that fewer than half of children 0 to 5 months in our sample were exclusively breastfed, more effort is needed to improve this health behavior. A previous study from Tanzania found that behavior change interventions that use mobile messaging and traditional interpersonal communication targeting fathers can benefit child health and disrupt harmful gender norms by engaging male participants and increasing their knowledge and improving their attitudes towards the importance of IYCN practices [19]. Similar findings emerged from a study in Western Kenya that found fathers and grandmothers who participated in nutrition dialogue groups supported mothers to improve infant feeding including dietary diversity [20]. Given the RISE II program’s use of husbands’ schools and grandmother groups, more focused interpersonal communication with these groups may help to address the negative associations detected in this study [21].

We found that children whose mothers were exposed to nutrition messages through radio, and health workers, and women’s group participation were more likely to have consumed adequate dietary diversity. These findings are consistent with previous studies that found exposure to nutrition messages through the radio and with health workers are positively associated with child nutrition practices [22,23]. Given the primarily rural setting and distance to health facilities for some communities in the Niger context, more effort may be needed to engage community health workers in IYCF counseling efforts [24]. Findings from previous studies have found that integrated SBC programs were most effective in improving nutrition related outcomes when led by community workers—likely a result of their intensive training in the relevant subject areas and understanding of the local context [25,26]. In addition, implementers may consider adopting an approach that leverages the influence of social networks, as previous research has found that having a neighboring mother participate in a nutrition behavior change intervention increased non-participants’ knowledge of appropriate nutrition behaviors as well [27]. This may include expanding the use of community videos to increase knowledge, attitudes, self-efficacy, and adoption of appropriate feeding practices, which has proven to be a promising approach in multiple settings [28,29] as well as leveraging the promising role of support groups to promote early initiation of breastfeeding and exclusive breastfeeding which has been shown to be more effective than peer counseling [30].

Previous studies have also considered the role of other gateway factors such as behavioral determinants and their association with IYCF practices. In Bangladesh, a cluster-randomized trial found an intervention aimed at increasing knowledge related to IYCF led to sustained knowledge about appropriate feeding practices 6–10 months after the intervention ended [31]. This is promising given the positive association with knowledge and minimum meal frequency identified in our results. A scoping review of observation, intervention, and effectiveness studies found that while several studies described norms, customs, and perceptions related to appropriate foods for young children, few explored the association with how they influenced IYCF practices [32]. Evaluations of norms-focused interventions reported improved child feeding practices but did not assess the impacts of the interventions on social norms. Our study finds that social norms play an important role in breastfeeding behaviors in Niger. Future research should consider exploring to what extent programs effectively shift these social norms. More evidence is also needed to understand what interventions would be most appropriate to address self-efficacy, which was positively associated with IYCF practices in our study.

Previous studies have found that men are central in health decisions in Niger [21,33] and culturally defined values and norms around women’s lack of agency and resources limit positive health decisions related to healthy feeding practices [34]. Consistent with the literature, we found that women who participated in three household decisions were more likely to initiate early breastfeeding [35–37]. Incorporating gender transformative approaches such as income generating activities or advanced homestead food production with agriculture, nutrition, and health behavior change and communication activities may provide additional support for women to generate resources and improve agency in decision making related to IYCF [38].

### Policy and practice and further research

This study provides evidence to strengthen implementation of continuum of care and SBC nutrition programs by identifying which behaviors are well suited for integrated programs and by determining which gateway behaviors can be used to more effectively sequence behavior change messages at strategic moments of interaction with the health system. The study draws from a broader program evaluation and did not conduct formative research to identify gateway factors and behaviors specific to the Niger context. Future studies aimed at understanding gateway factors and behaviors would benefit from conducting qualitative research to identify more precise and context specific measures.

### Limitations

The present study has limitations to consider. First, the cross-sectional nature of the study design meant it was not possible to empirically determine causal order between the gateway behaviors and nutritional outcomes. In addition, because we were unable to identify and isolate the gateway moment, the synergistic effects of the gateway moments, behaviors, and factors could not be determined. Nevertheless, the findings suggest that it would be useful to test if the observed behaviors occur around a gateway moment such as first pregnancy. In addition, our measures related to exposure to breastfeeding and child nutrition referenced the three months preceding the survey and therefore exposure could have occurred after the gateway behavior or nutritional outcome was initiated. However, given that the program had been ongoing since 2018, we hypothesize that respondents had been continuously exposed over the course of the program and the three-month recall serves as a proxy for longer term exposure to health information. Finally, contextual factors outside of those measured including drought and food security challenges while not measured may also be influencing the health and nutrition outcomes [39].

## Conclusion

In conclusion, the results of this study provide important insights to guide integrated SBC programmatic design. First, programs focused on increasing early initiation of breastfeeding should consider strengthening the normative environment for women to deliver in a health facility, given that women who delivered in a health facility at their last birth were more likely to adopt early initiation of breastfeeding. Similarly, increasing early and sustained use of ANC throughout a woman’s pregnancy may provide opportunities for health providers to introduce the importance of dietary diversity and minimum meal frequency for young children [40,41], establishing a path for improved nutritional outcomes. The identified gateway behaviors and factors also suggest that intervening before a woman becomes pregnant or early in her pregnancy might maximize the health impact on mothers and children. Different SBC strategies may be required based on the SBC determinants identified. Given the positive association of perceived social norms, effort should be directed at establishing positive social norms that support a woman’s efforts to adopt healthy IYCF practices particularly in light of the expanded marketing of breast milk substitutes through social media channels [42]. Finally, more evidence is needed to understand how these behavioral determinants motivate adoption of healthy nutritional behaviors and how contextual factors such as drought and food security contribute to these challenges.

## Supporting information

S1 Table Description of gateway factors for breastfeeding outcomes. (DOCX)

S2 Table Description of gateway factors for nutrition outcomes. (DOCX)

## Abbreviations

ANC: Antenatal care
CI: Confidence interval
IYCF: Infant and young child feeding
OR: Odds ratio
SBC: Social and behavior change
RISE II: Resilience in the Sahel Enhanced II
USAID: U.S. Agency for International Development
MWRA: Married women of reproductive age
