# Supplementary material for: Application of the integrated gateway model on child nutrition behaviors in Niger: An exploratory analysis
Source: PLoS One. 2024 Apr 1;19(4):e0297466. doi: 10.1371/journal.pone.0297466 (PMC10984541; doi:10.1371/journal.pone.0297466)
Supplement: S1 Table — (DOCX) [file pone.0297466.s001.docx]

Supplementary table 1: Description of gateway factors for breastfeeding outcomes (N=652)

|  | N (%) |
| --- | --- |
| Knowledge: State It is it healthy for a woman to give only breast milk for the first 6 months (reference: greater than 6 months or don’t know) | 465 (71.3) |
| Attitude: Agree if baby is exclusively breastfed for 6 months he/she is less likely to be sick (reference: disagree or neutral) | 579 (88.8) |
| Self-efficacy: Giving a baby food and liquids when he/she is 6 months of age is not difficult at all (reference: somewhat not difficult or very difficult) | 368 (56.4) |
| Perceived norms: State People in this community think is it healthy for a woman to give her baby only breast milk for the first 6 months (reference: greater than 6 months or don’t know) | 384 (58.9) |
| *Exposure to breastfeeding messages in last 3 months (reference: no exposure)* |  |
| Radio | 42 (6.4) |
| Health worker | 226 (34.7) |
| Community event | 83 (12.7) |
| *Gender-decision making* |  |
| Decides alone or jointly with partner (for purchases, visits, health seeking) (reference: partner decides) | 97 (14.9) |
| *Woman’s group participation (reference : no participation)* |  |
| Participated in group | 116 (17.8) |
