# Supplementary material for: Application of the integrated gateway model on child nutrition behaviors in Niger: An exploratory analysis
Source: PLoS One. 2024 Apr 1;19(4):e0297466. doi: 10.1371/journal.pone.0297466 (PMC10984541; doi:10.1371/journal.pone.0297466)
Supplement: S2 Table — (DOCX) [file pone.0297466.s002.docx]

Supplementary table 2: Description of gateway factors for nutrition outcomes (N=1,899)

|  | N (%) |
| --- | --- |
| Minimum meal frequency behavioral determinants |  |
| Knowledge: A child 6–23 months should eat 4 or more meals each day (reference: fewer than 4 or more meals) | 1,363 (71.8) |
| Attitude: Agree providing children four meals a day ensures they have strength (reference: disagree or neutral) | 1,854 (97.6) |
| Self-efficacy: Give child a meal four times a day is not difficult at all (reference: difficult or somewhat difficult) | 1,306 (68.7) |
| Perceived norms: Believes number of meals people in community think a child 6–23 months should eat each day is 4 or more (reference: fewer than 4 or more meals) | 675 (35.6) |
| Minimum dietary diversity behavioral determinants |  |
| Knowledge: Number of different types of food a child 6–23 months should eat a day is 4 or more (reference: fewer than 4 or more types of food) | 520 (27.4) |
| Attitude: Agree children who eat a variety of foods are less likely to get sick (reference disagree or neutral) | 1,814 (95.5) |
| Self-efficacy; Give child a minimum of five different types of food a day is not difficult at all (reference: somewhat difficult, or very difficult) | 450 (23.7) |
| Perceived norms: Believes number of different types of food people in the community think a child 6–23 months should eat a day is 4 or more (reference: fewer than 4 or more types of food) | 260 (13.7) |
| *Exposure to nutrition messages in last 3 months (reference: no exposure)* |  |
| Radio | 120 (6.3) |
| Health worker | 493 (26.1) |
| Community event | 218 (11.5) |
| *Gender-decision making* |  |
| Decides alone or jointly with partner (for purchases, visits, health seeking) (reference: partner decides) | 303 (16.0) |
| *Woman’s group participation (reference : no participation)* |  |
| Participated in group | 343 (18.1) |
